# N501Y mutation imparts cross-species transmission of SARS-CoV-2 to mice by enhancing receptor binding

**DOI:** 10.1038/s41392-021-00704-2

**Published:** 2021-07-27

**Authors:** Zubiao Niu, Zhengrong Zhang, Xiaoyan Gao, Peng Du, Jingjing Lu, Bohua Yan, Chenxi Wang, You Zheng, Hongyan Huang, Qiang Sun

**Affiliations:** 1 Laboratory of Cell Engineering, Institute of Biotechnology; Research Unit of Cell Death Mechanism, Chinese Academy of Medical Sciences, 2020RU009, Beijing, China; 2 Department of Oncology, Beijing Shijitan Hospital of Capital Medical University, Beijing, China

**Keywords:** Infectious diseases, Infection

**Dear Editor,**

According to the World Health Organization (WHO), as of March 8, 2021, the pandemic caused by severe acute respiratory syndrome coronavirus 2 (SARS-CoV-2) had infected more than 116 million patients with coronavirus disease 2019 (COVID-19) (https://covid19.who.int). The high infectivity of SARS-CoV-2 is largely attributable to the unique sequence composition of its spike (S) glycoprotein. During the process of viral infection, this glycoprotein can be processed into two fragments. The N terminal S1 fragment is responsible for receptor binding, and the C terminal S2 fragment promotes membrane fusion.^1^ SARS-CoV-2 is an RNA virus, and it has undergone frequent mutations, which have produced several variants over the past year. On top of this, the D614G mutation in the S glycoprotein has been shown to enhance viral infectivity.^2,3^ However, the functional implications of most mutations are largely speculative and not clear.

Recently, three highly prevalent variants have been reported. These are the variant Alpha (B.1.1.7, 501Y.V1), the variant Beta (B.1.351, 501Y.V2), and the variant Gamma (P.1, 501Y.V3). Even though multiple mutations have been identified in these three variants, the N501Y mutation in the receptor-binding domain (RBD) of S glycoprotein has been found to be the only shared mutation present on the S glycoprotein (Fig. 1a), suggesting a functional implication of the N501Y mutation in the transmission and prevalence of these three variants.

**Fig. 1:**
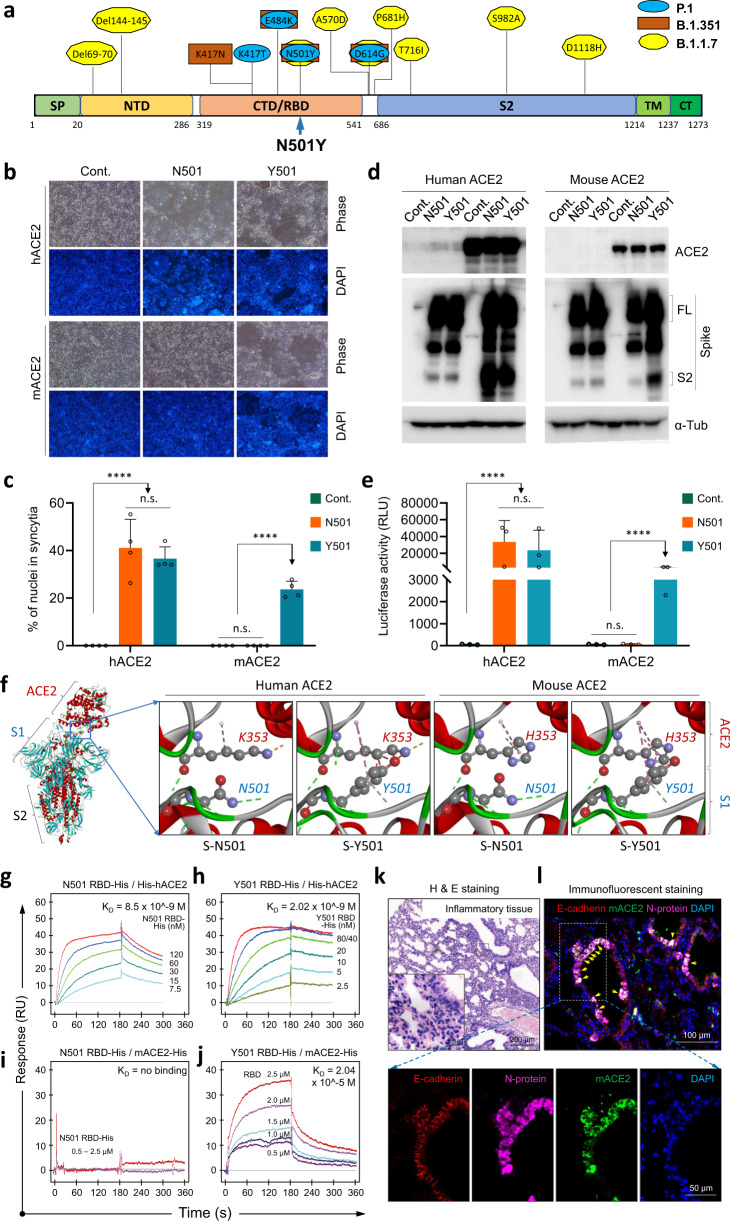
N501Y S glycoprotein binds to mouse ACE2 to mediate host entry and infection. **a** Schematic illustration of mutations in the spike protein of three SARS-CoV-2 variants: B.1.1.7, B.1.351 and P.1. Arrow indicates the N501Y mutation that was shared by the three variants in the receptor-binding domain (RBD) of S glycoprotein. **b**, **c** Representative images (**b**) and quantification (**c**) of syncytia formation upon expression of the indicated S glycoprotein in 293T cells expressing human ACE2 (hACE2) and mouse ACE2 (mACE2), respectively. Scale bars: 200 μm. Data are the mean ± SD of results from 4-5 fields (20x objective lens). ns not significant; ****p < 0.0001. More than three replicates were performed. **d** Expression of two types of spike proteins in the absence and presence of hACE2 and mACE2 in 293T cells 48 h post transfection by Western blot. FL full length. α-tubulin serves as the loading control. **e** Expression of the luciferase reporter in 293T-hACE2 and 293T-mACE2 cells upon infection of viruses pseudotyped with N501 or Y501 S glycoproteins as indicated. Data are the mean±SD of triplicate measurements. ns: not significant; ****p < 0.0001. **f** Three-dimensional structure modeling of the N501 and Y501 S glycoproteins in complexed with hACE2 and mACE2, respectively. The most left image shows the S glycoprotein trimer-ACE2 complex. Zoomed images show the interfaces of different complexes as indicated. The N501, Y501, Y353, and H353 residues are displayed in the style of scaled ball and stick with potential interaction bonds shown in dashed lines. **g**, **h** The Fc-tagged N501 (**g**) and Y501 (**h**) RBDs of SARS-CoV-2 S glycoprotein bind to His-tagged hACE2 with comparable affinities as determined by the biolayer interferometry analysis. **i**, **j** Biolayer interferometry analysis of the binding of His-tagged RBDs to His-tagged mACE2. Though N501 RBD hardly binds to mACE2, the Y501 RBD could effectively bind to mACE2 with a *K*_D_ of 2.04 × 10^−5^ M. The resulting data were fit to a 1:1 binding model. Each experiment was repeated independently twice with similar results. Five or six different protein concentrations were used to calculate the *K*_D_ values. **k** H&E staining of lung tissue from young (6-weeks old) BALB/c mouse infected with an authentic SARS-CoV-2 virus variant carrying N501Y mutation. Inflammation is evident. Scale bars: 20 and 200 μm, respectively, as indicated. **l** Immunofluorescent staining of mouse lung tissue infected with an authentic N501Y SARS-CoV-2 variant. Arrows indicate colocalization between ACE2 and S glycoprotein. Scale bars: 100 and 50 μm, respectively, as indicated

To test this idea, we first took advantage of the syncytium formation assay to explore the impact of N501Y mutation on S glycoprotein-mediated membrane fusion, an essential step in the process by which SARS-CoV-2 enters the host cell. As shown in Fig. 1b–d, the expression of S-Y501, referring to S glycoprotein with tyrosine (Y) as the 501^st^ amino acid, effectively induced syncytium formation in 293T cells expressing human angiotensin-converting enzyme 2 (hACE2) (293T-hACE2), the cellular receptor of SARS-CoV-2. However, S-Y501 seemed to be no more efficient than the S-N501, referring to S glycoprotein with asparagine (N) as the 501^st^ amino acid, in inducing syncytium formation (Fig. 1b, top panel, and Fig. 1c). Consistent with this, the production of S2 fragment, a functional domain mediating membrane fusion, was comparable in S-N501 and S-Y501 in the presence of hACE2 (Fig. 1d, left panel). The viruses pseudotyped with S-Y501 infected 293T-hACE2 cells at a level of efficiency comparable to that of S-N501-pseudotyped viruses (Fig. 1e). These results suggested that it is less likely that the N501Y mutation increases the infectivity of SARS-CoV-2 to human cells, though the N501Y mutation seemed to slightly alter the binding of S glycoprotein to hACE2, as indicated by structural analysis (Fig. 1f, left) and by affinity analysis on the Biacore^TM^ 3000 system (Fig. 1g, h).

The spread of the virus could be mediated by intermediate carriers, and the N501Y mutation was previously reported to be associated with mouse infection, but the causal link between them has not been established.^4^ We, therefore, hypothesize that N501Y mutation may be able to cross the species barrier and so cause infection in mouse cells. To test this idea, we expressed the Y501 S glycoprotein in 293T cells expressing mouse ACE2 (mACE2) (293T-mACE2), which resulted in marked syncytium formation, though the effect slightly less pronounced than in 293-hACE2 cells, while the N501 S glycoprotein had little effect (Fig. 1b, lower panel, and Fig. 1c). This was associated with an increased production of S2 fragment by Y501 S glycoprotein relative to N501 S glycoprotein in the presence of mACE2 (Fig. 1d, right panel). Furthermore, the Y501 S-pseudotyped viruses could effectively infect the 293T-mACE2 cells, in sharp contrast to N501 S-pseudotyped viruses, which failed to achieve any significant infection of the 293T-mACE2 cells (Fig. 1e). This spillover infection was likely a result of acquired interaction between the H353 of mACE2 and Y501 of S glycoprotein (Fig. f, right side), leading to a gain-of-function binding of Y501 S glycoprotein to mACE2, which was not observed when the 501^st^ amino acid of S glycoprotein was asparagine (N) (Fig. 1i and j). To further confirm the results of the in vitro cellular infection by pseudoviruses, we examined lung tissue sections from the wild-type BALB/c mice exposed to MASCp6, an authentic SARS-CoV-2 variant that carries the N501Y mutation.^4^ As shown in Fig. 1k, significant inflammation resembling typical pneumonia in humans manifested. Concomitantly, robust expression of viral N-protein was detected in cells along the airways, which was colocalized with ACE2 expression (Fig. 1l), indicating a successful infection of ACE2-positive cells by the N501Y variant.

Collectively, our results suggest that, unlike the most common D614G mutation, which has been shown to increase the infectivity of SARS-CoV-2 to cognate human target cells primarily by regulating the trimer stability of S glycoprotein,^3^ the N501Y mutation bestowed a gain-of-function involving the binding of S glycoprotein of SARS-CoV-2 variants to mouse ACE2, thus allowing host entry and cross-species infection. Consistent with our findings, Li et al. recently observed that the 501Y.V2 variants did not confer increased infectivity in human cells but rather displayed substantially enhanced infection of mouse cells.^5^ It’s therefore conceivable that mice may serve as an intermediate carrier to facilitate the transmission of N501Y SARS-CoV-2 variants, which warrants further epidemiological investigation. This idea, if confirmed, may shed light on the prevention of the spread of the N501Y variants. As such, mouse control may be an essential step.

It should be noted that the animal-mediated transmission of SARS-CoV-2 was only barely documented at present although multiple animals, such as cat, lion, ferret, had been reported as the susceptive hosts of SARS-CoV-2. This could be due to either the limited contacts with these animals, or the less effective infection of animal cells, or the ineffective replication in animal cells irrespective of effective infection, all of which may contribute to the ineffective animal-human transmission. It’s also possible that the human-human transmission is currently dominant over animal-human transmission in the spreading of SARS-CoV-2 and its variants, particularly in the countries/regions where the public health measures such as physical distancing, use of masks, personal hygiene, and isolation/quarantine were poorly implanted; thus, the animal-human transmission is undermined. All these possibilities are applied to the mouse-human transmission of the 501Y variants. Meanwhile, this study specifically investigated the effect of N501Y mutation on the infection of mouse cells via ACE2 binding while leaving out other co-existing mutations, which may miss the synergistic effects between them that may potentially contribute to mouse infection and subsequent spreading of the 501Y variants. Besides, though this study is attempting to make a connection between the N501Y mutation and mice-mediated SARS-CoV-2 spreading, it does not rule out the potential contribution of the N501Y mutation to the human-human transmission. As shown in the affinity assay, the binding of hACE2 to the S-Y501 is stronger than the hACE2_S-N501 interaction as shown by the lower K_D_ value (5.42 × 10^−9^ M vs 6.85 × 10^−9^ M), indicating a biophysical advantage of the S-Y501 over the S-N501. The enhanced receptor binding of the S-Y501 may potentially promotes the infectivity of the 501Y variants in a way undetected in the assays we utilized in this study, which warrants further investigations in the future.

## Supplementary information

3-supplemenary contents - clean

## Data Availability

All data generated during this study are included in this published article and its supplementary information files.
