# Supplementary material for: N501Y mutation imparts cross-species transmission of SARS-CoV-2 to mice by enhancing receptor binding
Source: Signal Transduct Target Ther. 2021 Jul 27;6:284. doi: 10.1038/s41392-021-00704-2 (PMC8313414; doi:10.1038/s41392-021-00704-2)
Supplement: Supplementary file 1 — 3-supplemenary contents - clean [file 41392_2021_704_MOESM1_ESM.docx]

Supplementary Materials for

**N501Y mutation is implicated in cross-species transmission of SARS-CoV-2 to mice by enhancing receptor binding**

Zubiao Niu^1†^, Zhengrong Zhang^1†^, Xiaoyan Gao^2†^, Peng Du^1†^, Jingjing Lu^1^, Bohua Yan^1^, Chenxi Wang^1^, You Zheng^1^, Hongyan Huang^2*^, Qiang Sun^1*^

† Equal contribution

* To whom correspondence should be addressed:

Qiang Sun

Email: [sunq@bmi.ac.cn](mailto:sunq@bmi.ac.cn)

Hongyan Huang

Email: [hhongy1999@126.com](mailto:hhongy1999@126.com)

**This PDF file includes:**

Materials and Methods

Supplemental reference

Figure S1 to S3

Tables S1 to S4

**Materials and Methods**

**Bioinformatics**

The 3D structure modeling of SARS-CoV-2 S glycoprotein was performed by the Modelling algorithm at SWISS-MODEL (https://swissmodel.expasy.org/) with the template of 7df4.pdb reported by Xu *et al* ^1^ from RSCB protein data bank (http://www.rcsb.org/).

**Cell culture**

The 293T, 293T-hACE2 and 293T-mACE2 cells were maintained in DMEM (MACGENE Tech Ltd., Beijing, China) supplemented with 10% fetal bovine serum (Kang Yuan Biol, Tianjin, China) and 1% Penicillin-Streptomycin (MACGENE Tech Ltd., Beijing, China). All cells were incubated with 5% CO2 at 37°C. The 293T-hACE2 and 293T-mACE2 were stable cell lines expressing hACE2 and mACE2, respectively.

**Constructs**

The codon-optimized SARS-CoV-2 S cDNA was synthesized at Genscript Biotech Corporation (Nanjing, China). The wild type S genes of SARS-CoV-2 and the Y501 mutation were cloned into pSecTag2-Hygro-A through seamless homologous recombination. Human ACE2 cDNA was cloned by PCR amplification from pMG-hACE2-Flag, which was a gift from Dr. Congwen Wei (Beijing Institute of Biotechnology), and inserted into pQCXIP-N1. The mouse ACE2 cDNA was purchased from Youbio (#G161730) and cloned into pQCXIP-N1 through seamless homologous recombination. Please find in supplementary tables for detail information on the constructs used in this study.

**Cell fusion**

For cell fusion assay, about 6 × 105 cells were plated per well in 6-well plate precoated with type I collagen (354236, BD Bioscience) and cultured for 24 h. Cells were then transfected with respective constructs by Lipofectamine LTX and Plus Reagent (Invitrogen, 1784283, USA) following the protocol provided. Images of 4 fields (20x objective lens) were taken on Hoechst-stained cells 48 hrs post transfection by Nikon microscope. Nucleus counting was performed by NIS elements AR software (Nikon, Japan). The fusion index (FI) was calculated as “% of nuclei in fused cells”.

**Western blotting**

Cells were lysed on ice with cold Radio-Immunoprecipitation Assay (RIPA) buffer containing phosphatase-protease inhibitors (CWBiotech, Beijing) for 20 min followed by ultrasound (power 40%, work 6 s, stop 9 s, 5 times in total). After being centrifuged at 12,000 rpm for 10 min, the supernatant was collected for SDS-PAGE electrophoresis followed by transferring onto the Polyvinylidene Fluoride (PVDF) membrane (0.2 μm, Millipore). The PVDF membrane, blocked with 5% skimmed milk for 1 hr at room temperature, was then blotted with primary antibodies in 5% BSA for 12 h at 4°C or 4 h at room temperature, followed by one-hour secondary antibodies at room temperature. The primary antibodies used: ACE2 (Proteintech, 1:1000, 21115-1-AP), SARS-CoV-2 spike (GeneTex, 1:2000, GTX632604), α-Tubulin (Proteintech, 1:1000, 11224-1-AP). The secondary antibodies used: anti-rabbit IgG HRP (CST, 1:3000, #7074), anti-mouse IgG HRP (CST, 1:3000, #7076).

**Pseudovirus production**

The mouse sarcoma virus（MSV）based SARS-CoV-2 S, and SARS-CoV-2 N501Y pseudotypes were prepared as previously described ^2^. HEK293T cells were co-transfected with an S encoding-plasmid, a Gag-Pol packaging construct (Addgene, 8449, USA) and the pQCXIP retroviral vector (Clontech, USA) expressing a luciferase reporter by using Lipofectamine LTX and Plus Reagent (Invitrogen, 1784283, USA) according to the manufacturer’s instructions. Cells were incubated for 6 hrs at 37℃ with transfection medium. Then transfection medium was changed with DMEM containing 10% Fetal Bovine Serum (FBS) was added for 48 h. The supernatants were then harvested and filtered through 0.45 μm membranes and then frozen at -80℃.

**Pseudovirus titration**

The titers of the pseudoviruses were calculated by the number of viral RNA genomes per mL of viral stock solution using RT-qPCR with primers that target LTR (Jiang, et al, 2020). Briefly, viral RNAs were isolated using TRIzol (Invitrogen, #15596026). One microgram of total RNA was converted into cDNA using TransScript® One-Step gDNA Removal and cDNA Synthesis SuperMix (Transgen Biotech, #AT311-02) according to manufacturer’s instruction. The quantitative PCR (qPCR) was performed on 15 ng of cDNA from each sample using SYBRGreen Real-time PCR Master Mix (TOYOBO, #QPK-201) based on the recommendations of manufacturer. pQCXIP-luciferase-vector was used to generate standard curves. The S-N501 and S-Y501 protein pseudotyped viruses were adjusted to the same titer (copies/mL or CCID50/mL) for the following experiments. Sense primer: 5’ - ATTCCCAATAAAGCCTCT - 3’, anti-sense primer: 5’ - GGTAGTCAATCACTCAGA - 3’

**Pseudovirus assay**

293T-hACE2 cells and 293T-mACE2cells were plated into 96 well plates at a density of 0.5×104 per well for 16 hr. About 1.15 × 104 copies of virus in the volume of 50 μL and 50 μL DMEM was added to the wells. After 12 hrs, 100 μL 10% FBS and 1% PenStrep containing DMEM was added to the cells. Following the 48 hr-infection, 100 μL One-Glo-EX (Promega, E6120) was added to the cells in equivalent culturing volume and incubated in the dark for 10 min prior to reading on an Enspire 2300 multilable reader (Perkin Elmer, USA). Measurements were done at least in triplicate and relative luciferase units (RLU) were plotted.

**Affinity assay**

The affinities between SARS-CoV-2 RBD recombinant proteins and human or mouse ACE2 were determined by multicycle kinetic analysis on the BiacoreTM 3000 system at 25°C. Human ACE2-Fc (KactusBiosystems, HM201, 031205) 8.0 μg/ml was captured on a CM5 chip (GE Healthcare, BR-1000-12, 10283568) handled with the Human Antibody Capture Kit (GE Healthcare, BR-1008-39, 10265753). Human RBD-His (GeneScript, P4FB003) at concentrations from 7.5 to 120 nM or RBD (N501Y)-His (Sino Biological, 40592-V08H82, MB14DE2661) from 2.5 to 80 nM in HBS-EP+ buffer was passed over the chip at a rate of 30 μL/min. The three-minute association time was followed by a three- or six-minute dissociation period. Mouse ACE2-His (Sino Biological, 50249-M08H, LC14AP1602) was directly immobilized on a CM5 chip by amine coupling. Human RBD-His or RBD (N501Y)-His at concentrations from 0.5 to 2.5 μM in PBS (pH7.4) was passed over the chip at a rate of 30 μl/min. Both the association and dissociation time were three minutes. The sensorgram curves were fitted to a 1:1 binding model (Langmuir) using BIAevaluation software, and kinetic constants were calculated.

**Immunostaining and imaging**

Tissue sections for lung tissues of 6 weeks old BALB/c mice infected with MASCp6, an authentic SARS-Co-V2 variant carrying N501Y mutation, were provided by Dr. Yusen Zhou from Beijing Institute of Microbiology and Epidemiology at Academy of Military Medical Sciences, China. Sections were routinely de-paraffinized with Xylene-Ethonal method followed either by H&E staining, or by microwaving-based antigen retrieval in citrate acid buffer for 15 min for immunofluorescent staining as described ^3^. Briefly, after 1 hour blocking in 5% BSA in TBS, samples were first stained with antibody against SARS-CoV-2 N protein (rabbit pAb from Sino Biological, 40143-R004) at dilution of 1:500 by Opal Multiplex tissue staining kit (Perkin Elmer, NEL791001KT) according to the standard protocol provided, the N proteins were eventually labeled with Cyanine 5 fluorophore. Slides were then incubated with mixed antibodies against E-cadherin (mouse mAb from BD Biosciences, 610181) and ACE2 (rabbit pAb from Proteintech, 21115-1-AP), followed by secondary antibodies of Alexa Fluor 568 anti-rabbit antibody (Invitrogen, A11036) and Alexa Fluor 488 anti-mouse antibody (Invitrogen, A11029). All slides were counterstained with DAPI to show nuclei and mounted with Prolong Gold antifade reagent (Invitrogen). Images were captured and processed by Ultraview Vox confocal system (Perkin Elmer).

**Statistics**

Data were expressed as means with standard deviations (SD). P-values were calculated using two-tailed Student's t-test from GraphPad Prism software, and P-values less than 0.05 were considered statistically significant.

**Supplemental reference**

1. Xu, C., et al., Conformational dynamics of SARS-CoV-2 trimeric spike glycoprotein in complex with receptor ACE2 revealed by cryo-EM. Sci Adv 7, (2021)

2. Jiang, X., et al., Bimodular effects of D614G mutation on the spike glycoprotein of SARS-CoV-2 enhance protein processing, membrane fusion, and viral infectivity. Signal Transduct Target Ther 5, 268-271 (2020)

3. Liang, J., et al., p53-dependent elimination of aneuploid mitotic offspring by entosis. Cell Death Differ 28, 799-813 (2021)

**Supplemental Figures**

**
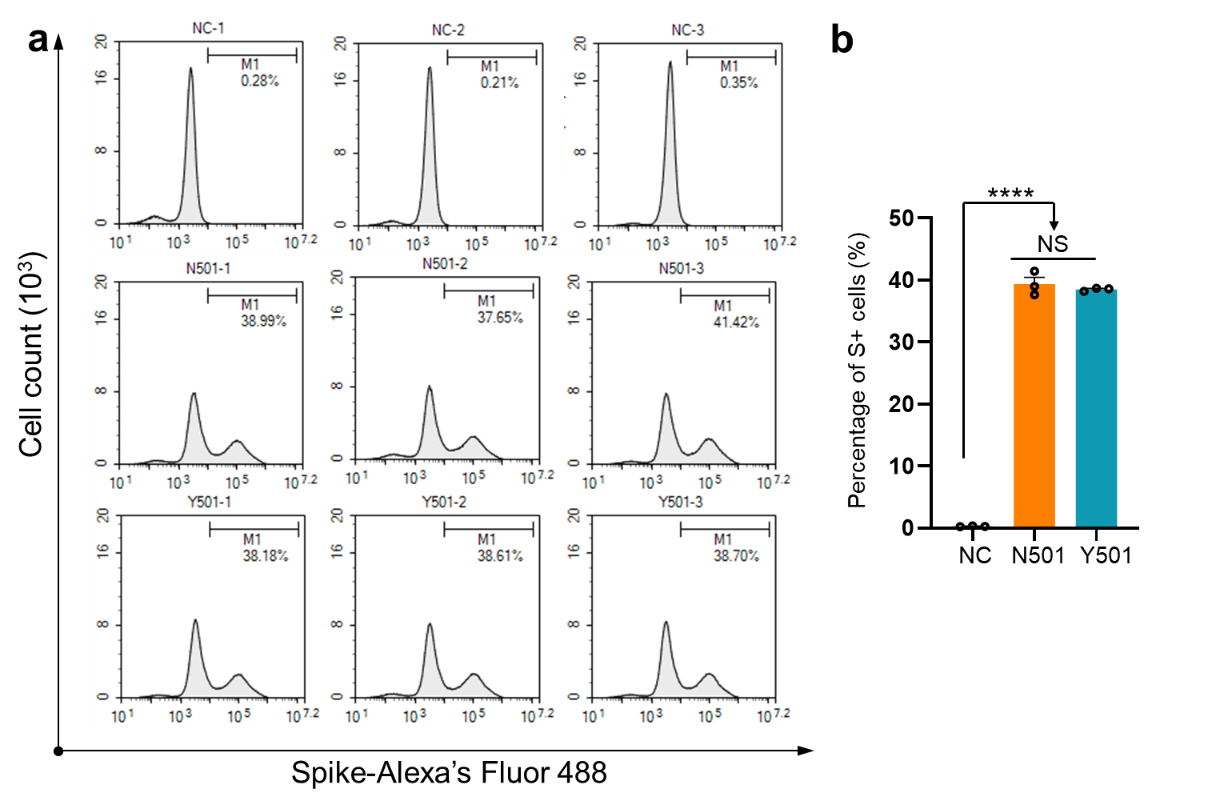
**

**Fig. S1 Flow cytometry analysis revealed no significant difference between wild type S-N501 protein and mutant S-Y501 protein expressed on the cell surface**. (**a**) Three replicates of each sample. M1: the gate of positive cells. (**b**): the percentage of S-positive (S+) cells. NS: no significant; ****: *p* < 0.0001.


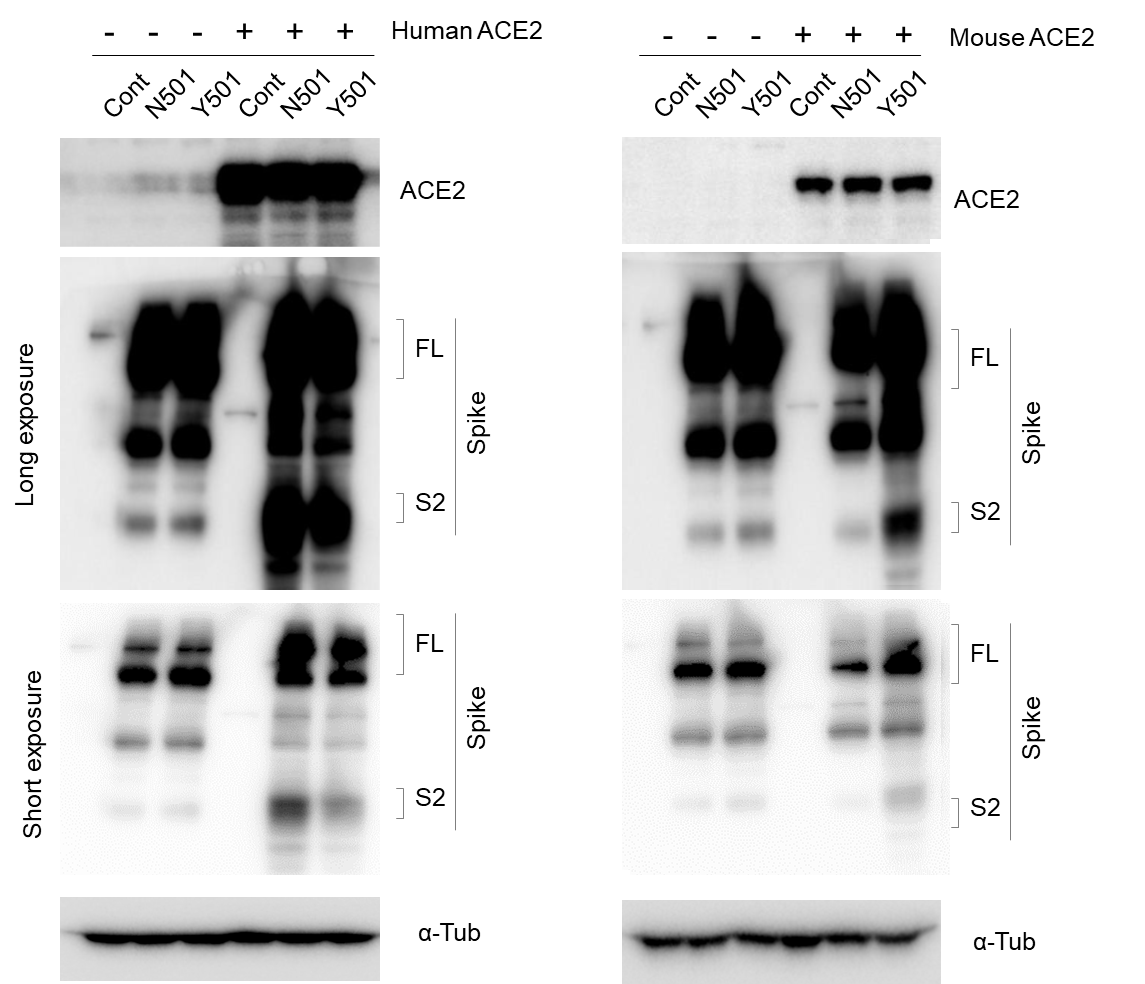


**Fig. S2 Comparable basal level expression of two types of spike proteins in the absence of ACE2 as detected by Western blot.** 293T Cells were collected 48 hours post transfection. FL: full length. α-tubulin (α-Tub) serves as the loading control. -: ACE2 deficient; +: ACE2 sufficient

**
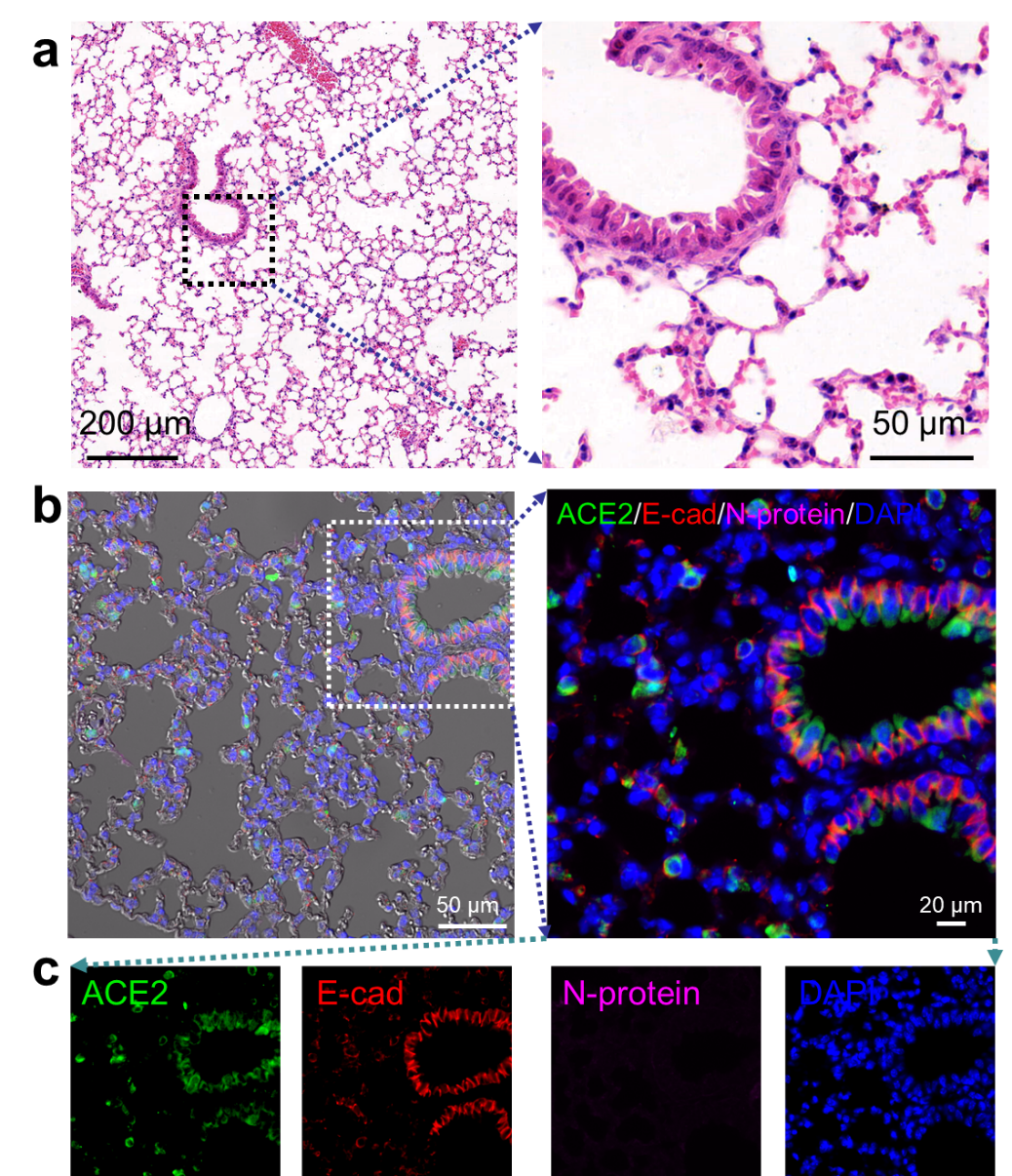
**

**Fig. S3 Staining of lung tissues from young (6 weeks old) BALB/c mouse infected with an authentic wild type SARS-CoV-2 virus (IME-BJ05)**. (**a**) H & E staining shows lung structures with no significant inflammation. Scale bars: 200 μm or 50 μm, as indicated. (**b, c**) no significant infection of mouse lung cells by wild type (N501 variant) SARS-CoV-2 as indicated by immunofluorescent staining with antibodies against ACE2, E-cadherin and N-protein. Scale bars: 100 μm or 50 μm, as indicated.

**Supplemental Tables**

Table S1. Information for constructs made in this study.

| Plasmids | Construct Method | Backbone | Site | DNA | Primer | Primer Sequence (5'→3') |
| --- | --- | --- | --- | --- | --- | --- |
| pSecTag2-COV2-S | Homologous Recombination | pSecTag2 Hygro A | *Xho* I | COV2-S | SARI-F1 | AGCTTGGTACCGAGCTCGCAGTGCGTCAATCTGACAACTCG |
|  |  |  |  |  |  |  |
|  |  |  | *Bam*H I |  | SARI-R1 | TTCGGGCCCTCCTCGAGCGGTGTAATGCAGCTTCACGC |
|  |  |  |  |  |  |  |
| pSecTag2-COV2-N501Y | Homologous Recombination | pSecTag2 Hygro A | *Xho* I | CoV2-N501Y-1 | HA-S1-F | ACGAAGCTTGGTACCGAGCTCG |
|  |  |  |  |  | N501Y-R | CCCACGCCGTATGTGGGCTGGAAGCCGTAGGAC |
|  |  |  | *Bam*H I | CoV2-N501Y-2 | N501Y-F | CAGCCCACATACGGCGTGGGCTATCAGCCTTAC |
|  |  |  |  |  | S2-R | TGAGTTTTTGTTCGGGCCCTCCTC |
| pQCXIP-EGFP-Luciferase | Cohesive End Ligation | PNL4.3.luc.R-E | *Eco*R V | Luciferase | Luc-F1 | ACGGTACCGCGGGCCACCATGGGTGGCGCGGCCGC |
|  |  |  |  |  |  |  |
|  |  |  | *Bam*H I |  | Luc-R1 | AGAGCCTGGACCACTGATCTAGGTCTCGAGCAATTTGGACTTTCCGCCC |
|  |  |  |  |  |  |  |
| pQCXIP-hACE2 | Cohesive End Ligation | pQCXIP-N1 | Sbf I | human ACE2 | ACE2-F1 | CATTGGAACGGACCTGCAGCCACCATGTCAAGCTCTTCC |
|  |  |  | Not I |  | ACE2-R1 | ATTATGATCTAGAGTCGCTCACTTGTCATCGTCATCCTTGTAGTCG |
| pQCXIP-mACE2 | Cohesive End Ligation | pQCXIP-N1 | Xho I | mouse ACE2 | Mouse-ACE2-F1 | CGGCCGCACCGGGATCTCGAATGTCCAGCTCCTCCTGGCTC |
|  |  |  | Pac I |  | Mouse-ACE2-R1 | CATGGTCTTTGTAGTCACGCGTCACCGGAAAGGAAGTCTGAGCATCATC |

Table S2. Constructs from Addgene.

| Name | Source | Cat. No | Inserts | Purpose |
| --- | --- | --- | --- | --- |
| pCMV-VSV-G | Addgene | 8454 | VSV-G | Envelope protein for producing lentiviral and MuLV retroviral particles |
| pUMVC | Addgene | 8449 | gag-pol | Packaging plasmid for producing MuLV retroviral particles. |

Table S3. Antibodies used in this study.

| Antibody | Company | Cat. No | Source | Type | Dilution |
| --- | --- | --- | --- | --- | --- |
| α-Tubulin | proteintech | 11224-1-AP | Rabbit | Polyclonal | WB 1:1000 |
| ACE2 | proteintech | 21115-1-AP | Rabbit | Polyclonal | WB 1:1000 |
| Spike [1A9] | Genetex | GTX632604 | Mouse | Monoclonal | WB 1:2000 |
| N protein | Sino Biological | 40143-R004 | Rabbit | Polyclonal | WB 1:200 |
| E-cadherin | BD Biosciences | 610181 | Mouse | Monoclonal | IF 1:500 |
| anti-rabbit Alexa Fluor 568 | Invitrogen | A11036 | Goat |  | IF 1:500 |
| anti-mouse Alexa Fluor 488 | Invitrogen | A11029 | Goat |  | IF 1:500 |
| Anti-rabbit IgG HRP | CST | 7074 | Goat |  | WB 1:3000 |
| Anti-mouse IgG HRP | CST | 7076 | Horse |  | WB 1:3000 |

Table S4. Cells used in this study.

| Cell lines | Source | Tissue Source | Cell type | Growth Properties | Culture medium |
| --- | --- | --- | --- | --- | --- |
| HEK 293T | maintained in the lab | human, kidney | epithelial cell | adherent | DMEM+10% FBS+1% Penicillin-Streptomycin |
| HEK-293T-hACE2 | constructed in the lab | human, kidney | epithelial cell | adherent | DMEM+10% FBS+1% Penicillin-Streptomycin |
| HEK-293T-mACE2 | constructed in the lab | human, kidney | epithelial cell | adherent | DMEM+10% FBS+1% Penicillin-Streptomycin |
